# Access to health care: the role of a community based health insurance in Kenya

**Published:** 2012-06-19

**Authors:** Judy Wanja Mwaura, Sathirakorn Pongpanich

**Affiliations:** 1 College of Public Health Sciences, Chulalongkorn University, Bangkok, Thailand

**Keywords:** Community-based health insurance, access, urban poor, informal settlements, Kenya

## Abstract

**Background:**

Out-of-pocket payments create financial barriers to health care access. There is an increasing interest in the role of community based health insurance schemes in improving equity and access of the poor to essential health care. The aim of this study was to assess the impact of Jamii Bora Health Insurance on access to health care among the urban poor.

**Methods:**

Data was obtained from the household health interview survey in Kibera and Mathare slums, which consisted of 420 respondents, aged 18 and above who were registered as members of Jamii Bora Trust. The members of Jamii Bora Trust were divided into two groups the insured and the non-insured.

**Results:**

In total, 17.9% respondents were hospitalized and women (19.6%) were more likely to be admitted than men (14.7%). Those in the poorest quintile had the highest probability of admission (18.1%). Those with secondary school education, large household size, and aged 50 and above also had slightly greater probability of admission (p<0.25). 86% of admissions among the insured respondents were covered JBHI and those in the poorest quintile were more likely to use the JBHI benefit. Results from the logistic regression revealed that the probability of being admitted, whether overall admission or admission covered by the JBHI benefit was determined by the presence of chronic condition (p<0.01).

**Conclusion:**

Utilization and take up of the JBHI benefits was high. Overall, JBHI favoured the members in the lower income quintiles who were more likely to use health care services covered by the JBHI scheme.

## Background

Inability to pay the out-of-pocket (OOP) expenditure required to access health services has been touted as one of the main impediments to access healthcare particularly for the poor and the vulnerable. OOP payments create financial barriers that prevent millions of people each year from seeking and receiving needed health services [1]. Household expenditure may account for up to 80% of total health expenditures due to high user charges (official or unofficial) in both public and private facilities (hospitals, clinics, diagnostics, medicines, and health care providers) and health insurance premium [2].

Given that low-income countries’ governments limited abilities to mobilize revenues, country and donor attention has turned to informal sector insurance mechanisms, such as Community Based Health Insurance (CBHI), as a way to improve financial protection, mobilize revenues, and improve the efficiency of out-of-pocket spending [3]. Under CBHI schemes, a group of people come together and voluntarily contribute small amounts to a common fund. Incase a member who contributes to the fund falls sick and needs treatment they can use the money in this fund. CBHI key principles include risk pooling and resource sharing [4].

Previous studies on CBHI and access to health reveal contradicting results. Some authors argue that CBHI schemes are a potential instrument of protection from the impoverishing effects of health expenditures for low-income populations [5], while others argue that the poorest in the community are excluded by CBHI [6]. Still, other studies say that the risk pool is often too small, that there are problems of adverse selection, the schemes depend heavily on subsidies, there are financial and managerial insufficiencies, and that the overall sustainability of schemes is questionable [7–9].

This study aimed to assess the impact of Jamii Bora Health Insurance (JBHI) scheme on access to inpatient health care. The authors of this paper are not aware of any other studies in Kenya that have documented the impact of CBHI on access to health care among members from urban informal settlements. Although, JBHI was established in 2001, this is the first formal study that examines the impact of the scheme on its members. The objective of this paper is to analyze the impact of community financing on access to health care using data from a household survey in urban informal settlements in Nairobi, Kenya. Kibera and Mathare slums in Nairobi were selected as the study sites for the following reasons: (a) a long experience with community-based health insurance schemes, (b) the large number of Jamii Bora Health Insurance members, and (c) the two slums are characterized by a high incidence of poverty and bad health conditions.

### Jamii Bora Health Insurance

The Jamii Bora Trust (JBT) Microfinance institute was established in 1999 and by the year 2010 it had over 110 branches, with over 277,000, spread all over Kenya. Its headquarter office is in Nairobi, Kenya. It is an organization of poor, self-employed informal workers who are engaged in small business and have no regular salaries. The goal of JBT is to help families, however poor, miserable, and hopeless to get out of poverty.

In 2001, JBT started the health insurance program, commonly referred to as Jamii Bora Health Insurance (JBHI), for its members whose goal is to improve its members’ access to quality health care. The membership has grown from 50 to over 13,000. The annual health insurance premium is KShs 1,200 (US$ 16) per member. The insurance covers the principle member and upto four children who are less than 18 years. The member can buy insurance for their spouses and/or pay an extra premium for any additional children beyond the four covered under the scheme. A member can either pay the full annual premium at one time or make weekly payments of KShs 30 (US$ 0.40) for 50 weeks, throughout the year. JBHI is the insurer and it has contracts with over 70 hospitals all over Kenya to provide inpatient services. All JBHI partner hospitals are either public or faith-based. JBHI covers only inpatient treatment costs and it has no co-payments, exclusions, nor cost restrictions. All members of JBT are eligible to join the health insurance on voluntary basis. However, it is compulsory for all members of JBT who have loans to pay health insurance. Any member may voluntarily choose to continue their membership even after they have finished their loan repayments [10].

JBHI is managed by a group of full employees under the JBT management. The JBHI management is responsible for determining the geographical coverage, setting the insurance premiums, and negotiates agreements with services provides e.g. ex-post settling of accounts. All the insurance premiums are deposited in a separate bank account.

In the event of illness, the member or their appointee presents themselves to their JBT branch office to get a letter that authorizes the partner hospital to provide inpatient health care services for the member. Members must only access health care services at partner hospitals. All monthly claims are sent to JBHI headquarter at the end of each month where the process of claim verification is done before payments are made. All hospital claims are settled within three months after receiving the invoices.

## Methods

This was a household survey that was carried out by the authors in 2010. A pre-test was carried out in September 2010 and the final survey took place in November 2010. A two-stage cluster sampling procedure was done using Jamii Bora Trust Databases. First, all the JBT members from both Kibera and Mathare were divided into two groups, insured and non-insured. The primary sampling unit was the member and simple random selection was used to select both insured and non-insured members until the sample population required for each area was enough. Visits were done to households and if the respondent was missing or refused to be interviewed the next nearest JBT member's household (insured or non-insured) was selected for interviewing.

JBT had a total of 277,092 members and of that, 32,132 were enrolled in the JBHI. Kibera and Mathare branches had approximately 15,901 and 12,246 members respectively [11]. Of these, insured members were 2,455 (15.4%) in Kibera and 2,117 (17.3%) in Mathare. Households were sampled in proportion to size and hence the sample population was Kibera branch 54% and Mathare branch 46%.

The probability of utilizing the health care services was the main determinant to calculate the sample size. Being insured or not insured was used as the main independent variable to divide the members of Jamii Bora Trust into two groups and compare health care utilization between them. This was chosen due to previous evidence showing that there was a difference in service utilization by insurance status. The sample size was 210 households for each group and 420 for the two groups. The sample size was estimated using the general formula of sample size determination in health studies [12].

Quantitative data was collected using a structured questionnaire with face-to-face interviews. A one-year recall period was used for self-reported hospitalization cases. A one-year recall period was chosen because the conditions were more serious and the events less frequent. In particular, details of illness and service utilization for each illness episode, e.g. disease or symptom, duration of illness, actions taken, hospitalization and length of stay, sources and amount spent, and satisfaction with care received were assessed. Key informant interviews were held with management of JBHI and the health service providers in order to get complementary information about the functioning, problems, and successes of JBHI. Separate Focus Group Discussions were held with insured and non-insured members of JBT. Using SPSS version 16, the data were entered immediately after completing the survey.

The study adopted Ronald Andersen Behaviour Model [13] to determine the study variables. This is a model of health care utilization that looks at three categories of determinants. First, predisposing characteristics represent the proclivity to utilize health care services where an individual is more or less likely to use health services based on demographics, position within the social structure, and beliefs of health services benefits. An individual who believes health services are useful for treatment will likely utilize those services. Second, are the enabling characteristics, which include resources found within the family and the community. Family resources comprise economic status and the location of residence and community resources incorporate access to health care facilities and the availability of persons for assistance. Third are need-based characteristics, which include the perception of need for health services, whether individual, social, or clinically evaluated perceptions of need.

The three categories of variables that determine service utilization derived from the Andersen Behaviour Model were employed as independent variables in the analysis. Predisposing variables included in the analysis were age, gender, marital status, education level, occupation, household size, and number of children. Enabling factors included were economic status, which was measured by household income. A monetary indicator was preferred because this was an urban population where cash income counts as wealth indicator. The per capita income was classified into 4 income quintiles of the total population. Quintile 1 was the poorest group and it was used as a comparison group. Income was variable of interest because the study sought to determine the extent to which demand for health care utilization was due to income level and paying ability. There are studies that have shown that the demand for health care is influenced by the ability to pay [14]. Hence income was used as an indicator for socio-economic status in this study. For the need factors, self reported presence of chronic condition was used as the indicator for health need required. Self reported utilization of health facilities was used as an indicator for service utilization.

### Ethical approval

The researchers sought ethical approval from University of Nairobi/Kenyatta National Hospital Health Research Ethical Committee and National Council for Science and Technology. Clearance from the Nairobi City Council Health Offices in Kibera and Mathare was also obtained. Oral and informed consent of the participants was obtained before the face-to-face interview was done.

## Results

The study findings show that there were no substantial differences in the demographic characteristics between the insured and non-insured respondents. Some notable differences were in the household characteristics, where the proportion of households in the moderate and higher income quintiles was higher among the insured (33.3%) than the non-insured (23.3%). There were more insured women (61.9%) in comparison to insured men (38.1%). The proportion of the insured was higher among the married (73.8%) than the unmarried (26.2%). Summary of descriptive statistics of the sample used in the analysis is shown in (Table 1).

**Table 1:** Descriptive Statistics of Study Sample

Socio-Demographic Data	Respondents		
	Insured N (%)	Non-Insured N (%)	Total N (%)
**Insurance Status**			
Insured	210 (100)	0 (0.0)	210 (100.0)
Non-insured	0 (0.0)	210 (100)	210 (100.0)
**Gender**			
Male	80 (38.1)	70 (33.3)	150 (35.7)
Female	130 (61.9)	140 (66.7)	270 (64.3)
**Age Group**			
18–35	71 (33.8)	98 (46.7)	169 (40.2)
36–49	84 (40.0)	84 (40.0)	168 (40.0)
50 and above	55 (26.2)	28 (13.3)	83 (19.8)
**Marital Status**			
Married	155 (73.8)	135 (64.3)	290 (69.0)
Unmarried	55 (26.2)	75 (35.7)	130 (31.0)
**Education Level**			
No formal school	20 (9.5)	11 (5.2)	31 (7.4)
Primary	71 (33.8)	83 (39.5)	154 (36.7)
Secondary and higher	119 (56.0)	116 (55.2)	235 (56.0)
**Number of Children**			
0–2	81 (38.6)	85 (40.5)	166 (39.5)
3–5	105 (50.0)	104 (49.5)	209 (49.8)
6 and above	24 (11.4)	21 (10.0)	45 (10.7)
**Household Size**			
1–4 (Small)	141 (67.1)	132 (62.9)	273 (65.0)
5 and above (Large)	69 (32.9)	78 (37.1)	147 (35.0)
**Religion**			
Protestant	132 (62.8)	113 (53.8)	246 (58.6)
Catholic	46 (21.9)	69 (32.9)	115 (27.4)
Islam	31 (14.8)	28 (13.3)	59 (14.0)
**Main Occupation**			
Casual workers	17 (8.1)	26 (12.4)	43 (10.2)
Public/Private employment	27 (12.9)	24 (11.4)	51 (12.1)
Business/trader	166 (79.0)	160 (76.2)	326 (77.6)
**Income Quintiles**			
1-Lowest	68 (32.4)	98 (46.7)	166 (39.5)
2-Low	72 (34.3)	63 (30.0)	135 (32.1)
3-Moderate	42 (20.0)	38 (18.1)	80 (19.0)
4-High	28 (13.3)	11 (5.2)	39 (9.3)

All descriptive statistics show percentages of total sample of insured and non-insured.

### 

#### Determinants of Enrolment in JBHI

The analysis indicated that the reasons for joining JBHI among the insured respondents differed in-line with the number of children the respondent had. Majority 65% of the insured respondents who had one or no children said that they joined JBHI because of the hard economy they are experiencing compared to 61% of respondents who had more than five children who said that they joined JBHI so that they could avoid out-of-pocket payments whenever a family member is admitted. Evidence on the association between household income status and JBHI enrolment indicates that individuals from the lower and lowest quintiles are more likely to be enrolled compared with those from the richest quintiles.

#### Utilization of Inpatient Health Care Services

Overall, the insured members were more likely to be hospitalized compared with non-insured members. Among respondents who reported having been hospitalized in the previous 12 months preceding the survey, 20.5% were insured and 15.2% were not insured. Majority (33.4%) of the insured were hospitalized during child delivery in comparison to (26.7%) of non-insured members. Among respondents who had been hospitalized for surgery treatment, (28.2%) were insured while (20%) who not insured.

A significant difference in probability of admission was observed by gender, marital status, income, and presence of chronic condition. Women (19.6%) were more likely to be admitted than men (14.7%), those in the lowest income quintile had a higher probability of admissions (18.1%), than those in the highest income quintile. Presence of chronic condition (29.9%), having secondary school education, large household size, and being aged 50 and above also had slightly greater probability of admission. Logistic regression results showed that, JBHI insured members are significantly more likely to be hospitalized than non-members. The probability of being hospitalized was higher among female, those with higher education, and those with chronic conditions (Table 2).

**Table 2:** Determinants of Hospitalization

Independent Variables	Respondents with at Least One Admission	*χ* ^2^	P-value
	n (%)		
**Insurance status**			0.161*
Insured	43 (20.5)	1.964	
Non-Insured	32 (15.2)		
**Gender**			0.203*
Male	22 (14.7)		
Female	53 (19.6)	1.619	
**Age Group**			0.395
18–35	29 (17.2)		
36–49	27 (16.1)		
50 and above	19 (22.9)	1.855	
**Marital Status**			0.297
Married	48 (16.6)	1.088	
Unmarried	27 (20.8)		
**Education Level**			0.200*
No School	7 (22.6)		
Primary	33 (21.4)		
Secondary and above	35 (14.9)	3.218	
**Number of Children**			0.611
0–2	26 (14.7)	0.987	
3–5	41 (19.6)		
6 and above	8 (17.8)		
**Household Size**			0.841
1–4 (small)	48 (17.6)	0.040	
5 and above (large)	27 (18.4)		
**Income Quintiles**			0.889
1	30 (18.1)	0.632	
2	26 (19.3)		
3	12 (15.0)		
4	7 (17.9)		
**Chronic Condition**			0.000**
No	35 (12.2)	19.298	
Yes	40 (29.9)		

* P<0.25

** P<0.01: Dependent variable: hospitalization in the past year (yes = 1; no = 0)

More frequent admissions were observed among the insured than those without insurance. Overall, 8% and 4% of insured and non-insured respondents respectively, had two or more admissions in the previous year. Being female, married, having higher education level, presence of a chronic condition, and smaller household size was associated with likelihood of having more than one admission in the previous year. Logistic regression results confirmed that the probability of being admitted more than once increased among those who were insured.

The insured members spend more days in hospital than the non-insured JBT members. It was observed that majority of the uninsured (43.2%) spend 1–5 days in hospital compared to the insured 57.3% in the same category. 34.5% and 65.5% of non-insured and insured members respectively stayed in the hospital for a period of 6–15 days, while 66.7% and 33.3% of non-insured and insured members respectively spent 16 or more days in hospital. On average non-insured members were hospitalized for 7 days with standard deviation of 9 days in comparison to the insured members who were hospitalized on average for 15 days with standard deviation of 28 days. Logistic regression results show that the insured are significantly more likely to spend more days in hospital than the non-insured members.

### Hospitalization and JBHI benefit take-up

Eighty six percent of formal admissions among the insured respondents were covered by the JBHI benefit. Notably, the insurance take-up was slightly lower among respondents in the higher income quintile (16.6%), male (27.0%), those without formal education (16.2%), and among the younger adults (37.9%). Those in the lowest income quintile were most likely to use the JBHI benefit when seeking inpatient health care compared to those in the highest income quintile, those with secondary school education level and above were more likely to use the JBHP benefit than those with less education, while women were more likely to use the benefit than the men. Overall, 87% of visits sought by the poorest income groups were covered by the JBHI benefit. The poor and those with chronic conditions had the highest utilization rate covered by the JBHI benefit.

## Discussion

This study aimed to determine the implementation of JBHI with regard to enabling access to health care for its members. This section discusses the methodological issues and the findings with regard to JBHI's impact on inpatient health care utilization for the urban poor.

Several strengths were noted in the use of the household survey. The survey data provides an accurate overview of social and household characteristics of residents of urban informal settlement. The scope of the survey, which included two large slums in Nairobi, the high participation, and the reliability of the information collected are some of the strengths noted in this study. Findings from the household survey can be used to form different panels to further explore other research questions. Although the scope of the survey was wide, the operational costs were low because of the small geographical area where the slums are located. However, access to the households was still difficult.

Although the study had an over-representation of women in the survey, analysis carried out showed that there was no difference in sex distribution by the insurance status and hence this did not affect the final results. The aim of the study was to compare the difference in inpatient service utilization among the insured and non-insured respondents. The sample was not drawn from the general population but from the same cluster (Jamii Bora Trust database) and they may not be independent from each other due to the similarities in socio-economic status, belief and/or even life styles. The small sample size for sub-group analysis was also a limitation in this study. The calculation of the sample size was based on the difference in service utilization among urban residents. The qualitative approach provided detailed explanation and hence an understanding of events that had been observed from the household survey. Limiting the study to only Jamii Bora Trust definitely limits the generalization of the study findings. Findings from this study can only be generalized for urban poor population but not for other urban or rural populations. Lack of information on the situation in the study population prior to enrollment in the JBHI is a major weakness of this study.

The JBHI has been implemented for over eight years and it seems to have overcome barriers that most CBHI encounter which lead to their failure. Its membership base has continued to grow annually. The main reason for this can be explained by its establishment within the JBT microfinance institution. Having the JBHI scheme as a benefit product to JBT members ensures that the members have access to loans and can grow their informal businesses and therefore can afford to pay the weekly premiums. Similar findings were observed among CBHIs that are within microfinance institutions, like SEWA in India where they tend to have high membership enrollment [15, 16].

The study found evidence that the number of children that a respondent had increased the likelihood of enrolling into JBHI. Respondents with six or more children were more likely to join JBHI to avoid out-of-pocket payments associated with medical expenses. These results show that enrollment in JBHI was associated with perceived financial risk of accessing health care and number of children that a member had.

Our finding that individuals in the low and lowest income quintiles are more likely to be enrolled in JBHI in comparison to others, is supported by previous studies, suggesting that inclusion of the poorest was dependent on the design and implementation features of the scheme [17]. JBHI collects weekly premiums throughout the year, which makes payment of premiums affordable even for the very poorest households. The finding that respondents’ perceptions of the quality of care provided by the contracted health care providers also influence the decision to enroll is similar to findings from previous studies [18, 19].

The comparison of insured and uninsured respondents through the household survey indicates that having health insurance leads to increased hospitalization amongst urban poor. In this study our findings suggest that the likelihood of hospitalization is positively associated with JBHI coverage. This result is supported by previous studies, which found that those who are insured are more likely to use inpatient health services [20, 21].

The presence of chronic condition was a significant predictor for the probability of an individual being admitted or admitted under the JBHP benefit in the previous year. Respondents with chronic conditions were 2.5 times more likely to be admitted than those admitted under the JBHP benefit in the previous year than those without chronic condition. This finding is consistent with the finding by Weisman and Jutting [22]. Long-term illness, such as HIV/AIDS and tuberculosis can cast the poor into a downward spiral of ever increasing indebtedness and hardship. Those in the higher income groups were less likely to be admitted or admitted under the JBHP benefit than those in the poor income groups. Hence income had a negative effect on the probability of admission or admission under JBHP scheme. Women were more likely to be admitted than men whether overall admission or admission covered by the JBHP.

Take-up of JBHI benefit for admission among the insured respondents was much higher (86%) than what other studies have observed. Previous studies have shown less CBHI take up of benefits up to 20% [23]. Several reasons could explain this observation. 1) JBHI does not require any co-payments at the point of seeking admission, 2) convenience access to health facilities could also be reason that encouraged and motivated the respondents to utilize the services, 3) the relatively high knowledge level among the insured about JBHI benefits and how to access the benefits, 4) the easy and straight forward procedure that the members have to comply with to access the free hospitalization, and 5) relatively high cost of care for hospitalization best explains the generally high take-up of JBHP benefit for hospitalization particularly among those in the lower income quintiles.

The take up of JBHP benefit was higher among the women, the married, and those with secondary education level or higher. This finding is in tandem with findings from Ekman[24] who found out that education levels are related with take up of health insurance programs in the developing world. Many low-income clients are unfamiliar with the concept of insurance program. The findings however disagree with [25] who found out that awareness by the government is more correlated to take up of health insurance policies compared to level of education of the urban poor. The results that show that most of urban poor use either the public and/or faith based hospitals for inpatient care, was supported by previous studies that found that the main reasons for this choice, was the type of services offered, the perceived quality of care and overall accessibility [26]. The finding that the insured spend more days in hospital than the non-insured is supported by previous which found similar findings.

## Conclusion

The study has shown that CBHI schemes such as JBHI can include the urban poor. Factors that facilitate inclusion of the urban poor include affordable premiums and positioning the scheme within a larger organization that addresses other needs of the poor.

Utilization and take up of the JBHI benefits was higher than most CBHI studies have shown and this could be attributed to the fact JBHI has no per capita financial capping and does not require any co-payments to access health care. Overall, JBHI favoured the members in the lower income quintiles who were more likely to use health care services covered by the JBHI scheme. Insured members reported higher use of hospitalization care than the non-insured. This confirmed that prepayment schemes and the pooling of risk could reduce financial barriers to health care among the urban poor.
